# The Interaction Effect of Gender and Residential Environment, Individual Resources, and Needs Satisfaction on Quality of Life Among Older Adults in the United Kingdom

**DOI:** 10.1177/2333721419878579

**Published:** 2019-09-24

**Authors:** Ben Chi-pun Liu, Dion Sik-yee Leung, Julia Warrener

**Affiliations:** 1 University of Hertfordshire, Hatfield, UK; 2 Caritas Institute of Higher Education, Hong Kong

**Keywords:** gender differences, formal and informal care, safety and security, social contact, use of time

## Abstract

**Objectives:** To examine the difference in gender and its impact on selected quality-of-life (QoL) domains of Social Production Function theory among older adults in England. **Method:** Based on an annual national adult social care service user survey conducted in the United Kingdom in 2016. QoL was assessed by a single-item construct, and independent variables were home design, access to information and local area, self-rated health, perceived pain/discomfort, perceived anxiety/depression, activities of daily living, use and satisfaction of formal and informal care, and demographic variables. **Results:** A total of 28,955 respondents aged 65+ years were interviewed. Multinomial logistic regression analysis found four interaction effects for predicting a very good/good QoL: (a) Female receiving non-co-residing informal care (odds ratio [OR] = 1.501, *p* < .01), (b) female feeling safe (OR = 1.499, *p* < .01), (c) female feeling satisfied with social contact with people (OR = 1.465, *p* < .05), and (d) female being helped in the use of time (OR = 1.370, *p* < .05). **Conclusion:** Findings suggest gender differences in QoL as men and women are heterogeneous with different health and disease patterns, health-/help-seeking behaviors, roles and responsibilities, and levels of resilience, needs, risks, and access and control resources. Practitioners should adopt a gender-specific assessment and personalized interventions to promote gender equality, empowerment, and long-term sustainable development for an aging society.

## Introduction

An exploration of gender differences should be considered as the basis for research because of the physiological and immunological differences between men and women (Ying, Pertrini, & Xin, 2013). Studies adopting gender perspective can provide insights into how differences across genders affect the illness course and progression, impact of risk factors of the disease, symptom profiles, and responses to treatment between men and women (Prata, Quelhas Martins, Ramos, Rocha-Gonçalves, & Coelho, 2016). A research paper on quality of life (QoL) was first published in a medical journal in 1974 (Bratt & Moons, 2015) and appeared in a Psychological Abstract in 1986 (Wingate, 2016). And QoL has become an important consideration ever since. Wingate (2016) then called for more new and clinically relevant QoL findings for publication.

Literature on QoL focuses mainly on its subjective and health-related components, such as physical and mental health, self-rated life expectancy, self-rated health, emotional functioning, social contact with people, relationship with primary carers, accessibility and social inclusion, financial well-being, personal development, religiosity, self-determination, and rights (Reed, 2007; Schalock, Verdugo, & Jenaro, 2005; Solomon, Kirwin, Van Ness, O’Leary, & Fried, 2010). QoL is a multidimensional concept evaluating a continuum of subjective intrapersonal dimensions and objective dimensions such as person–environment systems (Netuveli & Blane, 2008); however, a single-item global QoL scale can also be acceptable, sensitive, and valid compared to a multidimensional QoL scale (Yohannes, Dodd, Morris, & Webb, 2011). Studies on “ageing in place” indicate that the immediate environment such as the design of the home and accessibility of local area can significantly influence older adults’ QoL (van Leeuwen et al., 2014). This is because older people who score high on QoL are the ones enjoying independence, autonomy, social contact with people, and having a sense of security and attachment to their homes and communities (Wiles, Leibing, Guberman, Reeve, & Allen, 2011).

However, at that time, gender differences in QoL were rarely examined (Stöbel-Richter, 2013) as researchers usually treated research participants as gender-neutral persons, assuming no differences in the preferences and needs between men and women. In a cross-national community-based survey (Raggi et al., 2016) conducted in Finland, Poland, and Spain on QoL with 5,639 people, aged 18 to 50 years and 50+ years, it revealed that sociodemographic characteristics, social network, built environment, and chronic conditions such as disabling pain were the determinants of QoL. Another national adult social care survey conducted in the United Kingdom suggested something else and listed accessibility of information, design of the home, and accessibility to the local area being strong predictors of QoL (van Leeuwen et al., 2014). Martinez-Martin et al.’s (2012) study on 1,106 community-dwelling older men and women in Spain indicated that health, family, and finance were key determinants of QoL. Regrettably, none of the above community-based studies adopted a gender perspective to evaluate the similarities and differences concerning the determinants of QoL between men and women.

Even though gender differences were considered, most of the studies were conducted in clinical settings; and the gender of the participants was treated only as a moderator for examining the effects of independent variables on QoL (e.g., Chan et al., 2012). Furthermore, their research findings on the impact of diseases and outcomes of the medical treatment are not consistent from a gender perspective. Another case in point, the impact of a stroke or a transient ischemic attack on the QoL of male and female patients was variedly reported in the literature (Franzén-Dahlin & Laska, 2012). Research findings on gender differences in QoL in the general population samples are also mixed (Schnurr & Lunney, 2008). Although there were studies reported gender differences, for example, Michel, Bisegger, Fuhr, Abel, and The KIDSCREEN Group’s (2009) study found that female children and adolescents’ health-related QoL declined more than their male counterparts, those studies did not set out to validate the phenomenon of gender differences. It seems that the theoretical basis for examining the gender differences in QoL has not yet been developed. Therefore, Riedinger et al. (2001) suggested that future research on QoL should identify why gender differences exist. A gender perspective is useful in evaluating the impact of gender relations on QoL (Fadda & Jirón, 1999) because of the physiological, immunological, psychological, and sociodemographic and economic differences between men and women. And the differences can be subtle yet distinct.

In response to the gap in knowledge regarding gender differences in QoL, the current study adopted the theory of Social Production Function (SPF; Lindenberg & Frey, 1993; Ormel, Lindenberg, Steverink, & Verbrugge, 1999). SPF theory argues that the major goals for human being are physical well-being and social approval, and the fulfillment of basic need (i.e., needs satisfaction) is a necessary condition for good subjective well-being and effective functioning (Lindenberg, 1996; Liu, Dijst, & Geertman, 2016; Ormel et al., 1999). There are five interdependent instrumental goals serving as resources for achieving physical well-being and social approval, which are comfort, stimulation, affection, behavioral confirmation, and status (Wang, 2017). For example, older adults’ evaluation of life satisfaction is based on comfort (e.g., physiological needs, safety), stimulation (e.g., physical and mental arousing activities), affection (e.g., exchanging emotional support, feeling of caring), behavioral confirmation (e.g., social approval), and status (e.g., control over resources) as well as their individual resources and residential environment (Liu, Dijst, & Geertman, 2016; Ormel et al., 1999). The recognition of these goals can facilitate identifying what types of resources, care, and support required to enhance their overall well-being. Therefore, the SPF approach is recommended as a comprehensive and universal approach to understand the gender differences in stress and health (Steverink, Veenstra, Oldehinkel, Gans, & Rosmalen, 2011).

The United Kingdom is experiencing an aging population as 18% of people were aged 65 years and above in 2016 (Office for National Statistics [ONS], 2017). Older people are now living longer but with multiple chronic conditions. This subsequently gives the health and social care system the additional challenge that is to manage increasingly complex medical needs and psychosocial issues, which consequently affects older adults’ quality of life (QoL; Keefe et al., 2009). Informal care has been a core element of community care enshrined in legislation in the United Kingdom, such as the National Health Service (NHS) and Community Care Act 1990, and the Care Act 2014, to enhance the QoL of older adults living in community settings. Although community-based studies have been proposed as a new direction for QoL research (Kitchen & Muhajarine, 2008), limited studies examined the difference in gender and its impact on selected QoL items in the SPF theory domains among community-dwelling older adults. There are also few studies exploring the impact of both formal support services and informal care on QoL among older adults (Hellstrom, Persson, & Hallberg, 2004). In response to these above-mentioned gaps in research, the current study aimed to explore whether gender differences existed in selected QoL items among community-dwelling older adults in the United Kingdom and if so, to explain why. The current study hypothesizes the following:

1. Older female adults perceived a lower level of QoL than their male counterparts in the United Kingdom.
2. There were interaction effects between gender and residential environment, individual resources, and needs satisfaction in predicting a better QoL among older adults in the United Kingdom.

## Method

### Sampling

The Personal Social Services Adult Social Care Survey is an annual national survey measuring the effectiveness of formal social care services on service users aged 18+ years across England. All the formal social care service users in each local authority were invited to participate in the survey. For details of the sampling and data collection method, please refer to Health and Social Care Information Centre (HSCIC, 2016) and Adult Social Care Statistics Team (2016). Adult respondents were required to return their written consent form and completed questionnaire to their Local Authority so that answers were inputted into a database for analysis. The Social Care–Related Quality-of-Life (SCRQoL) questionnaire, which was based on the Adult Social Care Outcomes Framework (ASCOF), was used to measure how the use of formal care and support services influences QoL, including an individual’s control, personal care, food and nutrition, accommodation, safety, social contact, occupation, and dignity (Department of Health [DH], 2017; HSCIC, 2016; Netten et al., 2002). Employing stratified random sampling, a total of 73,165 service users responded to the survey in 2016 and the overall response rate was 35.7%. The current study is focused on analyzing the gender differences in QoL among those respondents aged 65+ years in receipt of community-based services.

### Data Analysis

Data analysis was conducted by using SPSS 25. All missing values were removed from the analysis. Chi-square test was employed to compare the gender differences in dependent variable (i.e., QoL) and independent variables among the older women and men. The dependent variable in the current study is nominal with three categories (i.e., Very good/good, Alright, Very bad/bad) and independent variables are dichotomous (i.e., binary), and multinomial logistic regression was therefore used. The “Very bad/bad” category of QoL was selected as the reference category to compare the probability of being in the “Very good/good” category of QoL. The Exp(*B*) (i.e., OR) of a coefficient indicates the probability of being in the comparison group compared to the probability of being in the reference category by an exposure to or absence of an independent variable. OR >1 means that a higher occurrence in the comparison group is due to the exposure of the independent variable. Interaction effects were created to examine whether the difference of gender has a significant effect on the independent variables in predicting a very good/good QoL.

The following variables are from the ASCOF and SCRQoL questionnaire:

1. Dependent variable—QoL was measured using a one-item construct, “Thinking about the good and bad things that make up your QoL, how would you rate the quality of your life as a whole?” The level of QoL was subjectively defined by the respondents after reviewing their current life quality and the answers were grouped into three categories: Score 1: “Very good/good,” Score 2: “Alright,” and Score 3: “Very bad/bad.” De Boer et al. (2004) argued that a single-item global QoL measure can also have good validity and excellent reliability.
2. Independent variables drawn from the ASCOF questionnaire were grouped in three SPF domains:
 2.1. Residential environment
  2.1.1. Home design: “How well do you think your home is designed to meet your needs?” —Score 1: My home meets my needs very well; Score 2: My home does not meet my needs well.
  2.1.2. Access to local area—Score 1: I can get to all the places in my local area that I want; Score 2: I find it difficult/am unable to get to all the places in my area that I want.
  2.1.3. Access to information and advice about support, services, or benefits—Score 1: Very easy to find; Score 2: Difficult/Very difficult to find.
 2.2.  Individual resources
  2.2.1. Self-rated health—Score 1: Very good/good; Score 2: Very bad/bad.
  2.2.2. Perceived pain or discomfort—Score 1: I have no pain or discomfort; Score 2: I have moderate/extreme pain or discomfort.
  2.2.3. Perceived anxiety or depression—Score 1: I am not anxious or depressed; Score 2: I am moderately/extremely anxious or depressed.
  2.2.4. Activities of daily living (ADL) including getting around indoors, getting in and out of bed or chair, feeding, bathing and washing, dressing, toileting, and dealing with finances, and paperwork. The overall score was grouped into Score 1: I can do it easily by myself; Score 2: I find it difficult/can’t this by myself.
 2.3. Needs satisfaction
  2.3.1. Formal care—including home care, day care, meals, short-term residential care, professional support, and equipment, or other community-based services provided by local authorities in England. Seven questions evaluating how the care and support services met respondents’ needs for control over daily life, clean and presentable appearance, food and drink, clean and comfortable home, safety, social contact with people, and how their time was spent. Score 1: Yes; Score 2: No.
  2.3.2. Informal care—two questions exploring whether the respondents received any practical support on a regular basis from co-residing carers and non-co-residing carers. Score 1: Yes; Score 2: No.
4. Demographic data including ethnicity (1: White; 2: Black and Minority Ethnic [BME] groups) and gender (1: Male; 2: Female).
5. Covariates—Levels of satisfaction with the care and support services: score ranging from 1: Extremely satisfied to 7: Extremely dissatisfied.

## Results

A total of 28,955 respondents aged 65+ years were selected for analysis in the 2016 survey. Table 1 shows that most of the older respondents were White (90.4%, *n* = 25,550) and 9.6% (*n* = 2,708) were from BME. About a third of the respondents (32.2%, *n* = 9,106) were male and 67.8% were female (*n* = 19,152). Slightly more than half of the respondents (54.9%, *n* = 15,895) rated their QoL as very good/good, 35.1% (*n* = 10,172) rated it as alright, and 10% (*n* = 2,888) rated it as very bad/bad. Fewer female respondents rated their QoL as very good/good than male respondents did (*p* < .01). More male and female respondents from BME groups perceived their QoL as very bad/bad compared to their White counterparts, respectively (male: BME = 13% vs. White = 10.4%, χ^2^ = 7.025, df = 2, *p* < .05; female: BME = 11.5% vs. White = 9.4%, χ^2^ = 9.359, df = 2, *p* < .01).

**Table 1.** Socio-Demographic Characteristics, QoL, and Overall Satisfaction With Formal Care.

(% within gender; % within ethnicity)	Male	Female	Total
Ethnicity			
White	8,198 (90.0; 32.1)	17,352 (90.6; 67.9)	25,500 (90.4; 100)
BME	908 (10.0; 33.5)	1,800 (9.4; 66.5)	2,708 (9.6; 100)
Total	9,106 (100; 32.2)	19,152 (100; 67.6)	28,258 (100; 100)^a^
Quality of life			
Very good/good	5,106 (55.1; 32.5)	10,735 (54.8; 67.5)	15,895 (54.9; 100)
Alright	3,201 (34.2; 31.5)	6,971 (35.6; 68.5)	10,172 (35.1; 100)
Very bad/bad	1,000 (10.7; 34.6)	1,888 (9.6; 65.4)	2,888 (10.0; 100)
Total	9,361 (100; 32.3)	19,594 (100; 67.7)	28,955 (100; 100)^b^*
Overall satisfaction with formal care	*M* = 2.39 (*SD* = 1.1)	*M* = 2.38 (*SD* = 1.1)^c^	

*Note*. QoL = Quality of Life; BME = Black and Minority Ethnic.

a χ^2^ = 2.338, df = 1, *p* = .066.

b χ^2^ = 10.537, df = 2, *p* < .01.

c *t* = .664, df = 16,340, *p* = .507.

* *p* < .05.

### Residential Environment and QoL by Gender

Table 2 reports that among those respondents who could not get to all places in their local area, there were more male respondents than female respondents rated their QoL as very bad/bad (*p* < .001). Similarly, there was a trend showing that there were more males than females rated their QoL as very bad/bad among those respondents whose home design did not meet their needs and who had difficulty in accessing information.

**Table 2.** Residential Environment and Quality of Life by Gender.

Quality of life				
(% within gender)	Very good/good	Alright	Very bad/bad	Total
My home design *does not* meet my needs well				
Male	2,145 (43.8)	2,023 (41.3)	726 (14.8)	4,894 (100)
Female	4,510 (44.2)	4,324 (42.4)	1,374 (13.5)	10,208 (100)^a^
*Cannot* get to all places in my local area				
Male	3,338 (48.5)	2,660 (38.6)	890 (12.9)	6,888 (100)
Female	7,874 (50.5)	6,021 (38.6)	1.708 (10.9)	15,603 (100)^b^*
*Difficult* to have access to information				
Male	2,787 (51.1)	2,032 (37.3)	631 (11.6)	5,450 (100)
Female	5,651 (50.9)	4,231 (38.1)	1,222 (11.0)	11,104 (100)^c^

a χ^2^ = 5.583, df = 2, *p* = .061.

b χ^2^ = 19.975, df = 2, *p* < .001.

c χ^2^ = 1.764, df = 2, *p* = .414.

*p < .001.

### Needs Satisfaction and QoL by Gender

Table 3 reveals gender differences in QoL among those who believed care and support services were not meeting their needs rated their QoL as very bad/bad QoL. There were more males than females believing formal care and support services could not help them in controlling their life (*p* < .05), in having a clean and comfortable home (*p* < .001), in maintaining social contact with people (*p* < .05), and in the way they spent their time (*p* < .01), so they rated their QoL as very bad/bad. Regarding the informal care utilization, there were also more males than females who received no practical support from co-residing carers and rated their QoL as very bad/bad (*p* < .01).

**Table 3.** Needs Satisfaction and Quality of Life by Gender.

Quality of life				
(% within gender)	Very good/good	Alright	Very bad/bad	Total
Care and support services *do not* help the respondent to have				
Control over daily life				
Male	455 (33.8)	510 (37.9)	381 (28.3)	1,346 (100)
Female	844 (34.6)	1,004 (41.2)	589 (24.2)	2,437 (100)^a^*
Clean and presentable appearance				
Male	575 (48.2)	444 (37.2)	174 (14.6)	1,193 (100)
Female	1,053 (48.3)	840 (38.5)	289 (13.2)	2,182 (100)^b^
Food and drink				
Male	1,019 (48.7)	788 (37.6)	286 (13.7)	2,093 (100)
Female	1,800 (51.3)	1,351 (36.9)	431 (11.8)	3,662 (100)^c^
Clean and comfortable home				
Male	1,178 (45.6)	1,021 (39.5)	387 (15.0)	2,586 (100)
Female	2,513 (48.7)	2,029 (39.3)	618 (12.0)	5,160 (100)^d^***
Feeling safe				
Male	884 (40.3)	896 (40.9)	412 (18.8)	2,192 (100)
Female	1,584 (39.2)	1,731 (42.8)	727 (18.0)	4,042 (100)^e^
Social contact with people				
Male	1,362 (45.5)	1,177 (39.3)	455 (15.2)	2,994 (100)
Female	2,829 (46.2)	2,490 (40.6)	807 (13.2)	6,126 (100)^f^*
The way you spend your time				
Male	1,333 (45.2)	1,168 (39.6)	449 (15.2)	2,950 (100)
Female	2,841 (46.6)	2,490 (40.9)	764 (12.5)	6,095 (100)^g^**
No practical support from co-residing carer				
Male	2,772 (56.1)	1,670 (33.8)	496 (29.6)	4,938 (100)
Female	6,829 (54.0)	4,636 (36.7)	1,180 (9.3)	12,645 (100)^h^**
No practical support from non-co-residing carer				
Male	2,604 (55.5)	1,544 (32.9)	546 (11.6)	4,694 (100)
Female	4,158 (56.3)	2,441 (33.1)	786 (10.6)	7,385 (100)^i^

a χ^2^ = 8.332, df = 2, *p* < .05.

b χ^2^ = 1.343, df = 2, *p* = .511.

c χ^2^ = 5.904, df = 2, *p* = .052.

d χ^2^ = 15.453, df = 2, *p* < .001.

e χ^2^ = 2.258, df = 2, *p* = .323.

f χ^2^ = 7.051, df = 2, *p* < .05.

g χ^2^ = 12.346, df = 2, *p* < .01.

h χ^2^ = 12.850, df = 2, *p* < .01.

i χ^2^ = 2.918, df = 2, *p* = .232.

* *p* < .05. **p < .01. ***p < .001.

### Individual Resources and QoL by Gender

Results show that there were more male respondents, who perceived a very bad/bad self-rated health (*p* < .01) and suffered from pain/discomfort (*p* < .001) and anxiety/depression (*p* < .001), graded QoL as a very bad/bad compared to female respondents did (Table 4). There were also more male respondents rated their QoL as very bad/bad than female respondents did. Similarly, there were more male respondents with a lower level of ADL also rated their QoL as very bad/bad than female respondents did (*p* < .001). Both male and female respondents from BME groups rated their health as very bad/bad compared with their White counterparts (male: BME = 29.5% vs. White = 21.4%, χ^2^ = 38.710, df = 2, *p* < .001; female: BME = 28.0% vs. White = 20.1%, χ^2^ = 86.993, df = 2, *p* < .001).

**Table 4.** Individual Resources and Quality of Life by Gender.

Quality of life				
(% within gender)	Very good/good	Alright	Very bad/bad	Total
Very bad/bad self-rated health				
Male	2,743 (42.9)	2,723 (42.5)	934 (14.6)	6,400 (100)
Female	6,073 (44.2)	5,918 (43.0)	1,759 (12.8)	13,750 (100)^a^*
Have pain or discomfort				
Male	5,024 (55.1)	3,127 (34.3)	971 (10.6)	9,122 (100)
Female	10,446 (54.7)	6,810 (35.7)	1,837 (9.6)	19,093 (100)^b^**
Have anxiety or depression				
Male	1,915 (41.7)	1,900 (41.3)	781 (17.0)	4,596 (100)
Female	4,335 (42.8)	4,304 (42.5)	1,485 (14.7)	10,124 (100)^c^**
Low level of ADL ability				
Male	1,712 (44.2)	1,518 (39.2)	645 (16.6)	3,875 (100)
Female	3,720 (44.6)	3,440 (41.2)	1,182 (14.2)	8,342 (100)^d^**

*Note.* ADL = activities of daily living.

a χ^2^ = 12.555, df = 2, *p* < .01.

b χ^2^ = 19.940, df = 2, *p* < .001.

c χ^2^ = 13.119, df = 2, *p* < .001.

d χ^2^ = 13.722, df = 2, *p* < .001.

* *p* < .05. **p < .001.

### Gender Differences in Predicting a Very Good/Good QoL

Multinomial logistic regression in Table 5 found four interaction items in predicting a very good/good QoL relative to a very bad/bad QoL, which are (a) Female × Receiving practice care from non-co-residing carer (OR = 1.501, 95% CI = [1.144, 1.968], *p* < .01), (b) Female × Feeling safe (OR = 1.499, 95% CI = [1.149, 1.956], *p* < .01), (c) Female × Satisfied social contact with people (OR = 1.465, 95% CI = [1.086, 1.978], *p* < .05), and (d) Female × Being helped in the way I spent my time (OR = 1.370, 95% CI = [0.999, 1.877], *p* < .05).

**Table 5.** Multinomial Logistic Regression Analysis on Quality of Life—Very Poor/Poor QoL as Reference Category.

Predictors	Gender	β	*SE*	Wald	OR	95% CI
Predictors with gender difference						
Support from a non-co-residing carer	Male	−0.254	0.180	1.987	0.775	[0.544, 1.104]
	Female	0.406	0.138	8.610	1.501**	[1.144, 1.968]
Feeling safe	Male	0.330	0.183	3.246	1.391	[0.971, 1.992]
	Female	0.405	0.136	8.907	1.499**	[1.149, 1.956]
Have social contact with people	Male	0.227	0.207	1.207	0.881	[0.881, 0.881]
	Female	0.382	0.153	6.230	1.465*	[1.086, 1.978]
Being helped in the way I spent my	Male	0.281	0.227	1.527	1.325	[0.848, 2.068]
	Female	0.315	0.161	3.829	1.370*	[0.999, 1.877]
Very good/good self-rated health	Male	2.111	0.320	43.451	8.259***	[4.408, 15.472]
	Female	2.099	0.226	86.085	8.159***	[5.237, 12.711]
No anxiety/depression	Male	1.309	0.177	54.449	3.701***	[2.614, 5.239]
	Female	1.042	0.128	66.014	2.836***	[2.205, 3.646]
Can get to all places in local area	Male	0.949	0.266	12.712	2.584***	[1.533, 4.354]
	Female	0.795	0.223	12.714	2.214***	[1.430, 3.427]
Have control and choice	Male	0.857	0.197	18.870	2.357***	[1.601, 3.470]
	Female	0.857	0.197	18.870	2.534***	[1.869, 3.436]
Good ADL functioning	Male	0.766	0.169	20.606	2.150***	[1.545, 2.993]
	Female	0.802	0.122	43.426	2.229***	[1.756, 2.830]
BME	Male	0.511	0.227	5.052	1.666*	[1.068, 2.601]
	Female	0.375	0.168	4.955	1.455*	[1.046, 2.023]
Home design meets my needs	Male	0.252	0.170	2.206	1.287	[0.923, 1.795]
	Female	0.105	0.368	0.081	1.110	[0.540, 2.283]
Home design not meet my needs	Female	−0.430	0.353	1.478	0.651	[0.326, 1.301]
Can access to information	Male	−0.154	0.220	0.491	0.857	[0.556, 1.320]
	Female	−0.095	0.169	0.316	0.909	[0.653, 1.266]
No pain/discomfort	Male	0.229	3.137	0.229	1.499	[0.958, 2.346]
	Female	0.184	0.208	0.184	1.088	[0.758, 1.561]
Have clean and presentable appearance	Male	0.366	0.200	3.350	1.295	[0.966, 1.735]
	Female	0.258	0.149	2.997	1.441	[0.974, 2.132]
Have food and drink	Male	−0.162	0.185	0.769	0.850	[0.592, 1.221]
	Female	−.142	0.134	1.119	0.868	[0.668, 1.128]
Have clean and comfortable home	Male	0.111	0.189	0.343	1.117	[0.771, 1.619]
	Female	−0.112	0.133	0.719	0.894	[0.689, 1.159]
Support from a co-residing carer	Male	−0.174	0.196	0.790	0.840	[0.572, 1.234]
	Female	0.235	0.149	2.468	1.265	[0.944, 1.695]
Satisfaction with care and support services		−0.647	0.042	236.495	0.524***	[0.482, 0.569]
Intercept	0.481	0.481	0.307	2.451		

*Note*. Dependent variable: quality of life (very good/good = 1, alright = 2, very bad/bad = 3). −2 Log likelihood = 10,003.211; chi-square = 2729.635 (df = 72)***; goodness-of-fit = 11,330.812 (df = 11,412), *p* = .703; Nagelkerke = .348; Cox and Snell = .295. QoL = quality of life; ADL = activities of daily living; BME = Black and Minority Ethnic.

* *p* < .05. **p < .01. ***p < .001.

## Discussion

### Results of Hypotheses Testing

Applying chi-square test for gender differences in QoL, the current study found that there were a statistically significant higher proportion of older men than older women perceived their QoL as very good/good, which confirmed the first hypothesis of this study. Results of multinomial logistic regression also found gender differences in four interaction items, that is, Non-co-residing informal care × Gender, Feeling of safety × Gender, Social contact with people × Gender, and The way how time was spent × Gender. This partially confirmed the second hypothesis of this study. The diversity of gender relations suggests that men and women are heterogeneous with different health and disease patterns, health-/help-seeking behaviors, roles and responsibilities, and levels of resilience, needs, risks, as well as access and control to resources.

### Gender Differences in Predicting QoL

A longitudinal Swedish study (Larsson, Kåreholt, & Thorslund, 2014) suggested that because of gender differences in mortality, older women have to depend on non-co-residing informal care or formal care when living alone in old age. The current study echoes this study indicating that the probability of grading QoL as very good/good QoL was 1.501 greater relative to a very bad/bad QoL among those older women with non-co-residing informal care. Our findings confirm previous research that informal care utilization is an influential factor to older adults’ physical health, control over daily life, and dignity (Hellstrom et al., 2004). In a way, obtaining the satisfaction of needs through utilizing formal and informal care is vital to improve QoL. Research found that women living with others have a lower QoL than those living with a spouse only (Henning-Smith, 2016). This may be because, as socialist-feminists argued, women are traditionally required to provide caregiving to their co-residing others and thus casting bio-psychological burdens and social restraint on female carers. Socialist feminism views that inequality and exploitation are socially constructed via the autonomous structures of gender race and class (Gordon, 2013). Gender differences in informal care utilization can be a result of differences in health conditions between the two genders. Those people receiving informal care tend to suffer more self-reported illnesses and a poorer QoL (Hellstrom et al., 2004), and therefore, informal care becomes a strong support network to older women who are having more physical and psychological health issues than men do. Although women tend to view receiving help as a loss of privacy and independence (Roe et al., 2001), literature suggests that older adults utilized both formal and informal care to fulfill their practical and emotional needs (e.g., Leung, Liu, Chow, & Chi, 2004; Liu, Cheng, & McGhee, 2001). This clearly reflects the notion of residual welfare in the United Kingdom. In fact, if older adults have substantial risk of falls, or if their living environment is not safe, the practical care provided by informal carers can facilitate a sense of safety for them (Bolin, Lindgren, & Lundborg, 2009). Practical support, such as food, provided by family members can also meet the dietary needs of elderly people (Jones, Duffy, Coull, & Wilkinson, 2009).

There are studies exploring the relationship between preference satisfaction and the fulfillment of basic human needs, such as safety and security on QoL (e.g., Costanza et al., 2007; Gabriel & Bowling, 2004). Our findings revealed that the probability of grading QoL as very good/good was 1.499 greater relative to a very bad/bad QoL among those older women who felt safe. This supported the literature that feeling a sense of safety is important to a good life quality (Costanza et al., 2007; Gabriel & Bowling, 2004). The sense of security in older women can be a result of, for example, safety from domestic violence and abuse, financial security after retirement, social security systems, and social and political stability. In Maslow’s hierarchy of needs, safety needs are put in a lower ranking; however, security was always rated more important than other needs in Maslow’s hierarchy for older people (Roszkiewicz, 2004). The current study echoes to Roszkiewicz’s findings that feeling safe was a strong predictor of QoL in elderly people, and particularly among older women. Therefore, health and social care service providers should safeguard elderly people as much as possible from any potential harm, risks, and adverse life events.

The current study suggested that the probability of grading QoL as very good/good was 1.465 greater relative to a very bad/bad QoL among those older women with good social contact with people. A meta-analytic review highlighted that social relationships are a significant risk factor for mortality across age (Holt-Lunstad, Smith, & Layton, 2010). A strong social tie and support network is effective in coping with challenges in aging because social support can fulfill the psychological and emotional needs of elderly people (Patterson & Veenstra, 2010). Drawing from their twin study, Agrawal, Jacobson, Prescott, and Kendler’s (2002) found a significant impact of genetic influence on relative support and confidence in females than in males. Cheng and Chan (2006) argued that women were socialized to take up the role of caretaker and tend to define their self-concept in terms of how they relate to their significant others. In other words, the process of socialization and biological heritage are key factors contributing to the gender differences in predicting an individual’s QoL by social contact.

Our results also show that the probability of grading QoL as very good/good was 1.370 greater relative to a very bad/bad QoL among those older women being helped in the way how they spent their time. The gender differences in the use of time can be explained by the social and cultural norms. Although literature suggested four types of time use, that is, paid work, unpaid work, self-care, and free time (Bittman & Wajcman, 2000), traditional gender-role attitudes expect women to take up a traditional gender role and motherhood, resulting in different patterns of time use between the two genders (Baxter, 2015). As a result, older women spend more time on household labor, family care, child-rearing, home-making, and care-giving than older men (Meggiolaro & Ongaro, 2015). As argued by socialist-feminists, capitalism and patriarchy have collectively and specifically oppressed unpaid women carers for producing free services to substitute or supplement formal social care (Ward-Griffin & Marshall, 2003). Although the Activity Theory and Continuity Theory highlighted the positive outcomes of active time use in aging, such as volunteering, multi-morbidity and lack of individual or environmental resources hinder older adults’ desire to participate in social leisure activities (Galenkamp et al., 2016). In contrast, the SPF theory suggests an integrative approach to understand how people obtain life satisfaction by achieving their universal needs via instrumental goals within their constraints (Wang, 2017). The constraint that older women in the current study faced was suffering from more health problems compared to older men. However, they still perceived a very good/good QoL due to the help from care and support services. Therefore, formal care may thus become a protective factor for QoL among older women who face the gender inequality in time use and adverse health conditions.

### Limitations of the Study

The current study has several limitations. This analysis was based on a cross-sectional survey, so it was difficult to identify causal relationships among variables, which may affect the generalizability. The dataset released for public access does not include enough data for understanding how the socioeconomic and demographic data contributed to the gender differences in QoL in older adults. Therefore, caution should be taken in interpreting and generalizing the current findings. Using a predictive statistical model to conceptualize gender differences based on the three SPF domains, that is, residential environment, individual resources, and needs satisfaction, may not effectively generate rich understanding of gender influences in the men’s and women’s subjective feelings and experiences of multidimensional QoL. Nevertheless, the current study is based on a U.K. territory-wide annual survey, interviewing around 73,000 adult social care services users. Its quantitative analysis and findings can still provide insights to geriatric and gerontological practitioners and policymakers for developing integrated care and support services to enhance the QoL of frail and vulnerable older adults. In addition, QoL was measured by a single-item scale, which may be unable to reflect the comprehensiveness and complex concept of QoL. Yet, literature reports that the single-item QoL measure is also valid and reliable in clinical and community-based research compared to multi-question measure (De Boer et al., 2004; Yohannes et al., 2011).

## Conclusion

The current study identified gender differences in four determinants of QoL, that is, feeling a sense of safety, social relationships, occupation of time, and use of non-co-residing informal care. There are limited studies about gender differences in the changes of QoL over time (Schmidt et al., 2005). The future direction of QoL research should therefore use a validated theoretical framework such as SPF theory to identify and explicate the gender differences in QoL among older adults. In addition to culturally sensitive perspective, health and social care practitioners working with older service users should also adopt a gender perspective to identify the unique needs of each individual and tailor-made personalized care plans for them. They should be aware of the potential gender bias in the assessment of QoL and avoid gender stereotype or assume a sameness of men and women (Obaidi & Mahlich, 2015). As the focus of assessment and intervention should be on the service user rather than the problem (Felton, 2005), a gender-sensitive approach to co-producing integrated care by the helping professionals, service users, and informal carers can promote the QoL among older adults. That development is a good start to promote gender equality, a likely lodestar to lead the way to gender empowerment, and responsive, long-term sustainable development for our aging society.
